# Manual dexterity in school-age children measured by the Grooved Pegboard test: Evaluation of training effect and performance in dual-task

**DOI:** 10.1016/j.heliyon.2023.e18327

**Published:** 2023-07-15

**Authors:** Valerio Giustino, Antonino Patti, Luca Petrigna, Flavia Figlioli, Ewan Thomas, Vincenza Costa, Luigi Galvano, Jessica Brusa, Domenico Savio Salvatore Vicari, Simona Pajaujiene, Daniela Smirni, Antonio Palma, Antonino Bianco

**Affiliations:** a Sport and Exercise Sciences Research Unit, Department of Psychology, Educational Science and Human Movement, University of Palermo, Palermo, Italy; b School of Medicine, Department of Biomedical and Biotechnological Sciences, Anatomy, Histology and Movement Science Section, University of Catania, Catania, Italy; c Department of Coaching Science, Lithuanian Sports University, Kaunas, Lithuania; d Department of Psychology, Educational Science and Human Movement, University of Palermo, Palermo, Italy

**Keywords:** Manual dexterity, Fingers dexterity, Hand movement, Fine motor skills, Motor coordination, Finger tapping test, Counting test, Dual-task, Musculoskeletal system, Neuromuscular system

## Abstract

**Background:**

Manual dexterity is the ability to manipulate objects using the hands and fingers for a specific task. Although manual dexterity is widely investigated in the general and special population at all ages, numerous aspects still remain to be explored in children. The aim of this study was to assess the presence of the training effect of the execution of the Grooved Pegboard test (GPT) and to measure the performance of the GPT in dual-task (DT), i.e., during a motor task and a cognitive task.

**Methods:**

In this observational, cross-sectional study manual dexterity was assessed in children aged between 6 and 8. The procedure consisted of two phases: (1) the execution of five consecutive trials of the GPT to evaluate the training effect; (2) the execution of one trial of the GPT associated with a motor task (finger tapping test, GPT-FTT), and one trial of the GPT associated with a cognitive task (counting test, GPT-CT) to evaluate the performance in DT.

**Results:**

As for the training effect, a significant difference (p < 0.001) between the five trials of the GPT (i.e., GPT1, GPT2, GPT3, GPT4, GPT5) was detected. In particular, we found a significant difference between GPT1 and GPT3 (p < 0.05), GPT1 and GPT4 (p < 0.001), and GPT1 and GPT5 (p < 0.001), as well as between GPT2 and GPT4 (p < 0.001), and GPT2 and GPT5 (p < 0.001).

As for the performance in DT, no differences between the best trial of the GPT (i.e., GPT5) and both the GPT-FTT and GPT-CT was found.

**Conclusion:**

Our findings suggest that the execution of the GPT in children has a training effect up to the third consecutive trial. Furthermore, the administration of the GPT in DT does not affect GPT performance.

## 1 Introduction

The upper and lower limbs have the fundamental function of allowing the carrying out of the activities of daily living (ADLs) [1]. While the lower limbs are responsible for carrying out gross motor skills such as walking, running, or jumping, the upper limbs are instead specialized not only in gross motor skills such as throwing, catching, or lifting, but also in fine motor skills such as writing, typing, or cutting, and more generally in manipulating any object with precision [1]. Indeed, fine motor skills describes the ability to perform and control small hand movements to carry out a task with precision using eye-hand coordination (i.e., visuo-motor integration), spatial-temporal processing (i.e., spatial-temporal integration), and task-related muscle strength accuracy [2,3].

It is widely recognized that high level of both gross and fine motor coordination in children is associated with higher academic achievement, better cognitive performances, and also it positively influences the practice of physical activity [[4], [5], [6], [7], [8], [9]].

Based on this evidence, the importance of exploring manual dexterity during the developmental age is crucial. Manual dexterity, including manual and fingers dexterity, is defined as the ability to manipulate objects using the hands and fingers in order to perform movements with precision and in a timely manner for a specific task [[10], [11], [12]]. Manual dexterity develops from early childhood and is perfected during development and is essential for several ADLs such as tying shoelaces or handwriting [13]. Children with poor fine motor coordination show an inadequate control of precision grip/lift between the index finger and the thumb for holding an object. Since several ADLs, such as writing, involves a control of precision grip the study of manual dexterity in school-aged children needs to be extensively investigated [14].

The school age is a crucial stage for motor development in children due to various factors including motor learning and a greater functional autonomy of body segments [15]. The visuo-motor integration and the spatial-temporal integration, abilities required for the execution of manual dexterity tasks, are fundamental for motor control and motor learning [16].

Measuring manual dexterity is one of the goals of research in this field [12,17]. In research and clinical practice, the measurement of both manual and fingers dexterity represents a neuromotor function assessment of the hand and the fingers, respectively [11]. The several assessments for manual dexterity available measure the speed and/or the quality of the movements of hand/fingers [11]. Among the latter, the Grooved Pegboard test (GPT) is widely used and consists in manipulating with thumb and index finger the pegs that have a ridge and in inserting each of them into the holes of the pegboard, each of which oriented in a different way.

Although scientific literature shows that manual dexterity measured by the GPT is widely investigated in the general and special population at all ages, numerous aspects still remain to be explored in children. For instance, to the best of our knowledge, there are no norm scores, and there are no studies on training effect, and on performance in dual-task (DT). Previous studies have explored factors or predictors for manual dexterity performance [16,18]. Considering that during ADLs children are often faced with situations that require the simultaneous processing and/or execution of motor and cognitive tasks [19], manual dexterity performance in DT in this population should be studied further.

In a recent study by Petrigna et al. (2020), authors investigated the presence of a training effect of the GPT and the performance of the GPT during a motor task and a cognitive task in a sample of young adults [20]. Authors detected a training effect up to the 4th consecutive trial. Furthermore, they found that the execution of the GPT in DT was significantly slower than the trials performed singularly both during the motor task and cognitive task.

Based on these premises, the purpose of this study is to explore manual dexterity in children measured by the GPT. In particular, we aimed to assess the presence of the training effect of the execution of the GPT and to measure the performance of the GPT in DT, i.e., during a motor task and a cognitive task. The novelty of the study was to explore these factors for manual dexterity performance in typically developing children.

We hypothesize that the consecutive repetition of trials of the GPT leads to a training effect. Furthermore, our hypothesis is that the execution of the GPT in DT should show lower performance than performing the GPT alone.

## 2 Materials and methods

### 2.1 Study design

This research is an observational, cross-sectional study in which manual dexterity was assessed in a sample of children aged between 6 and 8 and attending the first and second grade of a primary school.

Firstly, the procedure and purpose of the study were explained to the head teacher. Following the accession of the school, the study was presented to the parents of the children of the first and second grade, informing them that participation would be on a voluntary basis after filling out an informed written consent.

The study was conducted in accordance with the recommendations of the Declaration of Helsinki for the involvement of people in research and was approved by the Bioethics Committee of the University of Palermo (n. 88/2022).

### 2.2 Participants

The a priori sample size analysis was computed using G*Power software 3.1.9.2 (Heinrich Heine University, Düsseldorf, Germany) which showed that a total sample size of 21 participants (f = 0.25, α = 0.05, power = 0.80) was required for the first procedure phase of this study which consisted of 5 repeated measures, and a total sample size of 28 participants (f = 0.25, α = 0.05, power = 0.80) was required for the second procedure phase which consisted of 3 repeated measures.

For this study, a total of 40 children, including 22 boys and 18 girls, were recruited from a primary school in Palermo, Sicilia (Italy). To be enrolled, participants had to meet the following inclusion criteria: (1) be aged between 6 and 8 years; (2) attend the first or second grade; (3) being right-handed verified through the Edinburgh Handedness Inventory [21,22]; (4) both sedentary and active; (5) both male and female. Participants were excluded in case of: (1) upper limbs injuries; (2) neuromuscular/neurological diseases; (3) mental disorders; (4) drug therapy that could have affected neuromuscular or cognitive function.

As participants were minors, parents provided an informed written consent to permit to their children's participation in the study.

### 2.3 Study procedure

Data collection took place in a school room from 8:30 a.m. to 1:30 p.m. (i.e., during school time) and was carried out by the same two investigators. We assessed all participants during school hours in order to minimize time-of-day effect on manual dexterity performance. Participants were called one by one from the classes to which they belonged, and each participant was tested only once. The duration of the test session was approximately 15 min.

The procedure used is the one developed by Petrigna et al. (2020) and consisted of two phases: (1) firstly, the execution of five consecutive trials of the GPT with a 1-min rest between trials to evaluate the training effect; (2) after a 3-min rest, the execution of one trial of the GPT associated with a motor task (finger tapping test), and one trial of the GPT associated with a cognitive task (counting test) with a 1-min rest between trials to evaluate the performance in DT [20]. During both phases the time for completing each trial of the GPT was recorded.

### 2.4 Grooved Pegboard test

In this phase, each participant performed five consecutive trials of the GPT (i.e., GPT1, GPT2, GPT3, GPT4, GPT5) with a 1-min rest between trials to evaluate the training effect.

Each participant was asked to sit in a chair behind a desk on which the GPT was placed.

Before administering the GPT, each participant familiarized with the test filling only the first top row.

The GPT (Lafayette Instrument Company, Inc.; Lafayette, IN, US) is composed of a 25-hole board, in a 5 × 5 grid, with randomly keyhole orientation and pegs with a key along one side. The test consists of placing the pegs according to the keyhole orientation, with the right hand and one by one, into the 25 holes filling the holes line by line from left to right and from top to bottom in the shortest possible time.

During the execution of the GPT each participant was asked to keep the left hand on the desk. Furthermore, it was not allowed to pick up the pegs that fell during the test, but each participant had to continue the test by picking up another peg.

The time (s) to complete each trial of the GPT (i.e., from when the participant took the first peg to when the last peg was inserted in the grid) was recorded using a stopwatch.

### 2.5 Grooved pegboard test in dual-task

In this phase, each participant performed one trial of the GPT associated with a motor task (finger tapping test), and one trial of the GPT associated with a cognitive task (counting test) to evaluate the performance in DT.

The finger tapping test (FTT) consists of tapping the index finger of the left hand against the desk repeatedly.

The counting test (CT) consists of counting from zero to ten and from ten to zero, and so on again without stopping, until the grid was filled of pegs.

The execution and the assessment of the GPT was the same as previously described and the two trials in DT (i.e., GPT-FTT and GPT-CT) were administered in a random order among the participants.

### 2.6 Statistical analysis

Descriptive statistics were carried out to present data as means and standard deviations.

The Shapiro-Wilk test was performed to evaluate data distribution.

The Friedman's test was executed to investigate differences between the five trials of the GPT. The Dunnett's test was conducted to carried out multiple comparisons between the five trials of the GPT. These analyses were performed to assess the presence of the training effect of the execution of the GPT.

The Friedman's test was also used to analyse differences between the best trial of the GPT, and both the GPT in DT (i.e., GPT-FTT and GPT-CT) with the Dunnett's test for multiple comparisons. These analyses were carried out to measure the performance of the GPT in DT.

Statistical analyses were performed using GraphPad Prism 7 (GraphPad Software Inc., San Diego, CA, USA) with p-value set significant at <0.05.

## 3 Results

Descriptive statistics of participants’ characteristics are reported in Table 1. Descriptive statistics of the performance of the five consecutive trials of the GPT and of the GPT in DT are reported in Table 2.

**Table 1:** Descriptive statistics of participants’ characteristics.

N (b, g)	Age (Years)	Height (cm)	Weight (kg)
40 (22, 18)	6.88 ± 0.46	123.49 ± 7.39	28.87 ± 8.99

**Legend.** N, number of participants; b, boys; g, girls.

**Table 2:** Descriptive statistics of performance of the five consecutive trials of the GPT, and performance of the GPT in DT.

	GPT1	GPT2	GPT3	GPT4	GPT5	GPT-FTT	GPT-CT
Time (s)	98.84 ± 30.68	95.52 ± 34.36	91.74 ± 36.80	88.09 ± 29.95	86.65 ± 27.47	87.47 ± 20.69	92.29 ± 52.39

**Legend.** GPT, Grooved Pegboard Test; DT, Dual-Task; GPT1, first trial of the GPT; GPT2, second trial of the GPT; GPT3, third trial of the GPT; GPT4, fourth trial of the GPT; GPT5, fifth trial of the GPT; GPT-FTT, GPT associated with the finger tapping test; GPT-CT, GPT associated with the counting test.

The Shapiro-Wilk test showed that data were not-normally distributed.

Data showed that the best trial of the five consecutive trials of the GPT was identified in the last trial (i.e., GPT5) in which participants required 86.6 ± 27.5 s to complete the test.

**Fig. 1:**
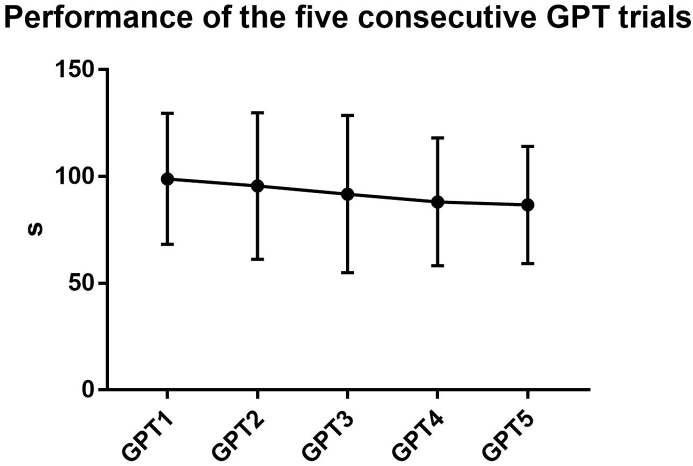
Performance of the five consecutive GPT trials. Legend. GPT, Grooved Pegboard Test

Regarding the assessment of the training effect, the Friedman's test detected a significant difference (p < 0.001) between the five trials of the GPT (i.e., GPT1, GPT2, GPT3, GPT4, GPT5). As shown in Fig. 1, the Dunnett's multiple comparisons test showed a significant difference between GPT1 and GPT3 (p < 0.05), GPT1 and GPT4 (p < 0.001), and GPT1 and GPT5 (p < 0.001). A significant difference was also found between GPT2 and GPT4 (p < 0.001), and GPT2 and GPT5 (p < 0.001).

**Fig. 2:**
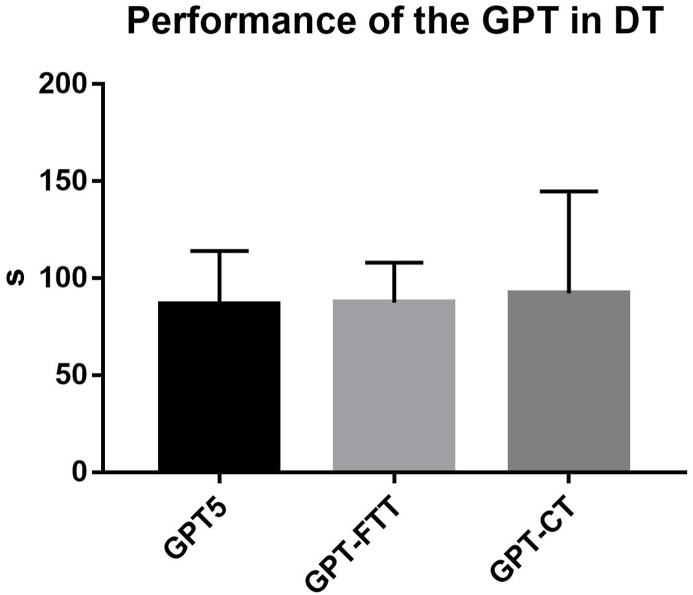
Performance of the GPT in DT Legend. GPT, Grooved Pegboard Test; DT, Dual-Task.

As concerns the measurement of the performance in DT, the Friedman's test showed no differences between the best trial of the GPT (i.e., GPT5) and both the GPT-FTT and GPT-CT (Fig. 2).

## 4 Discussion

This study aimed to explore manual dexterity in children through the GPT. In detail, the following two aims have been investigated: (1) to assess the training effect of the execution of the GPT; (2) to measure the performance of the GPT in DT, i.e., during a motor task (GPT-FTT) and a cognitive task (GPT-CT).

Our hypothesis was partially confirmed. In fact, although we detected a training effect of the execution of the GPT, no differences were found between the performance of the GPT in DT and the execution of the GPT alone.

Although several research groups have investigated manual dexterity in children with disorders/disabilities [[23], [24], [25], [26], [27], [28]], the scientific literature presents very few studies in which school-age children have been evaluated [16,29]. Hence, this study aims to provide new knowledge in this field focusing on the study of training effect and on the influence of a DT on manual dexterity performance. Indeed, previous studies have reported the importance of manual dexterity in children. The findings by Nobusako et al. (2018) showed that manual dexterity is a predictor of visuo-motor temporal integration ability, regardless of children's age [16]. Obeid & Brooks (2018) demonstrated a relationship between manual dexterity and language ability in school-age children [29].

Regarding the first aim of the study, our results have detected the presence of training effect during the execution of the GPT trials. In particular, no significant differences were found from trial 3 to trial 5. In fact, multiple comparisons results indicate a training effect up to the third consecutive trial. As a matter of fact, differences with the same significance level (p < 0.001) were found between GPT1 and both GPT4 and GPT5, as well as between GPT2 and both GPT4 and GPT5. A difference with a lower significance level (p < 0.05) was also found between GPT1 and both GPT3. These results are partially in line with those previously published by Petrigna et al. (2020) in which authors detected a training effect up to the fourth consecutive trial in young adults [20]. Thus, a training effect also exists in children, but this occurs earlier (up to 3rd consecutive trial) than in young adults (up to 4th consecutive trial). Indeed, training effect is an adaptation of the neuromuscular system to the repetition of a task that led to performance enhancement [30]. This can be explained by the fact that the repeated practice produces a repeated exposure of the brain to the same task that induce to learn the appropriate motor commands for that task [31]. Moreover, trial-by-trial errors play a fundamental role for the acquisition of task-specific motor commands through internal models learned from error-feedback [32]. The training effect we found could be related to the neurobiological mechanisms involved for the specificity of this task that include a visuo-manual coordination and a prediction of the shape and the size of the object to be grasped before contact with it in order to perform a quick and precise movement [13,17]. Our results suggest that this task required three consecutive trials of training effect in children. The difference we detected in the occurrence of the training effect between our sample (i.e., up to 3rd consecutive trial in children) and that of the previous study (i.e., up to 4th consecutive trial in young adults) [20] reflects the different motor skill learning mechanisms and post-training processes existing between children and adults [33]. As a matter of fact, gains in motor skill slow as they develop further over multiple practice sessions, and this progression of motor skill learning seems to depend on appropriate stabilization and consolidation processes [33,34]. Moreover, previous research in which the authors administered motor tasks, involving the 5-item explicit finger-to-thumb opposition sequence, showed faster performance improvements in children than in adults [35,36]. Similar results were found in grapho-motor task comparing children and young adults [33].

As concerns the second aim of the study, we found no differences between the best trial of the GPT (i.e., GPT5) and both the GPT-FTT and GPT-CT. These results are in contrast with some researches which showed a worsening of manual dexterity performance in DT condition [20,37,38]. However, none of these studies involved children. Our results could be explained by the type of dual task selected which, although appropriate for the age of the participants, may not require a high motor/cognitive effort to affect performance. A recent work investigated brain regions activated in both right‐handed adults and children during a finger tapping test under differing task through functional magnetic resonance imaging (fMRI) [39]. Considering that children have an underdeveloped motor system and lower experience in the execution of the task, they should show a greater activation of brain areas than adults. However, authors’ findings revealed that the brain areas activated during the finger tapping test are the same regardless of age [39]. Nonetheless, among the differences, authors found that children showed a greater activation in left primary sensorimotor cortex, and adults showed a greater activation in right supplementary motor area [39]. This result could provide a rationale for the absence of differences between the execution of the GPT-FTT and the GPT alone that we found in children, contrary to the study by Petrigna et al. (2020) where a lower performance in the GPT-FTT compared to the GPT alone was detected in adults. Considering the GPT-CT, counting up to 10 adding “1” and backwards is an ability that all children at this age are capable of [40]. Notwithstanding the choice of cognitive task was adequate, scientific evidence suggests that children from populations with languages that have simpler rules governing counting exhibit a greater counting [40]. Therefore, we assume that the cognitive task adopted may have been too easy for Italian children of this age.

## 5 Conclusions

In conclusion, our findings suggest that the execution of the GPT in children has a training effect up to the third consecutive trial. Furthermore, the administration of the GPT in DT (using the counting up to 10 and backwards as cognitive task, and the finger tapping test as motor task) does not affect GPT performance.

### 5.1 Strengths and limitations

Among the strengths of the study, it should be mentioned the sample size power achieved, the homogeneity of the sample, and the novelty of the study. The limitations concern the fact that we have not taken into consideration the socio-cultural level, the economic background, and the academic achievements of the children recruited.

## Author contribution statement

Valerio Giustino: Conceived and designed the experiments; Analyzed and interpreted the data; Wrote the paper.

Antonino Patti; Flavia Figlioli; Ewan Thomas: Analyzed and interpreted the data.

Luca Petrigna: Conceived and designed the experiments; Analyzed and interpreted the data.

Vincenza Costa; Luigi Galvano: Performed the experiments.

Jessica Brusa; Domenico Savio Salvatore Vicari; Simona Pajaujiene; Daniela Smirni: Contributed reagents, materials, analysis tools or data.

Antonio Palma; Antonino Bianco: Conceived and designed the experiments.

## Data availability statement

Data will be made available on request.

## Additional information

No additional information is available for this paper.

## Declaration of competing interest

The authors declare that they have no known competing financial interests or personal relationships that could have appeared to influence the work reported in this paper
